# Nutritional Supplementation and Enhanced Antioxidant Function by Dietary Intake of Selenoneine and Other Selenium Compounds in Red Seabream *Pagrus major*

**DOI:** 10.1007/s10126-023-10215-6

**Published:** 2023-07-18

**Authors:** Yutaro Shimokawa, Kanako Abe, Mami Ohura, Manae Yamamoto, Hitoshi Ando, Takuma Tohfuku, Michiaki Yamashita, Masakazu Kondo

**Affiliations:** National Fisheries University, Shimonoseki, Yamaguchi 759-6595 Japan

**Keywords:** Selenium, Selenoneine, Intake, Antioxidant, Fish

## Abstract

Selenoneine, 2-selenyl-*N*_*α*_, *N*_*α*_, *N*_*α*_-trimethyl-_L_-histidine, is the major organic selenium compound in marine fish. To characterize biological antioxidant function of selenoneine in fish, the accumulation of selenoneine and other selenium compounds, i. e., sodium selenite and selenomethionine, in the muscle and other tissues of red seabream. We reared red seabream by feeding of 1% dry pellet containing of sodium selenite, selenomethionine, or selenoneine of body weight twice a day for 4 weeks. After that, we replaced to 1% of normal commercial dry pellet of body weight twice a day for 1 week from the selenium supplementation, and tissue distribution of total selenium was determined. Selenium supplementation with selenoneine, selenomethionine, and sodium selenite enhanced selenium accumulation in the white muscle, kidney, and hepatopancreas in comparison with the control group. By the dietary intake of selenoneine, total selenium concentrations were increased in the white muscle, heart, kidney, spleen, hepatopancreas, brain, and blood cells in a dose-dependent manner during the trials after 2 weeks. Dietary intake of selenoneine as well as sodium selenite and selenomethionine reduced oxidation–reduction potential (ORP). Selenoneine concentrations in the white muscle and blood cells were accumulated for 4 weeks by the selenoneine intake, whereas selenoneine concentration was not elevated by the intake of selenomethionine and sodium selenite, suggesting that tissue selenoneine levels might be derived from only selenoneine-containing diet. The uptake factor of selenoneine from the artificial feed containing selenoneine was calculated to be 0.0062 in the white muscle and 4.0 in the blood. The half-life of total selenium in the blood cells and white muscle were estimated to be 60 days in the white muscle and 30 days in the blood.

**Supplementary Information:**

The online version contains supplementary material available at 10.1007/s10126-023-10215-6.

## Introduction

Selenium is an essential micronutrient for animals (Combs and Combs 1986; Himeno and Imura 2000; Imura and Naganuma 1991). Selenium has several physiological functions as selenocysteine for the redox enzymes, such as glutathione peroxidase (Brigelius-Flohé 1999; Rotruck et al. 1973), thioredoxin reductase (Mustacich and Powis 2000), thyroid hormone deiodinases (Arthur et al. 1993), and selenoprotein P (Burk and Hill 1994). The antioxidant function of selenium plays protective roles in 50 human diseases, including prostate, lung, and intestine/colon cancers, immunodeficiency and heart diseases (Ghose et al. 2001; Kiremidjian-Schumacher and Roy 2001; Salonen et al. 1982). Selenium deficiency caused myopathy, exudative diathesis, and pancreatic degeneration to animals and birds (Combs and Combs 1986). Muscle dystrophy by selenium deficiency was also reported in carp and rainbow trout (Bell et al. 1984; Combs and Combs 1986; Gatlin and Wilson 1984; Poston et al. 1976). It was also shown that feeding juvenile rainbow trout a plant protein-based diet without added selenium may be detrimental to the antioxidant status of the fish body (Fontagné-Dicharry et al. 2015). Tohfuku et al. (2021) reported that the intake of selenoneine imparted the antioxidant properties of selenium to amberjack. The biological antioxidant functions of selenium-containing compounds are considered to be dependent on the chemical form of molecular species. “Burnt meat” is a problem in aquaculture and fisheries of pelagic ocean fish, such as tuna, yellowtail, and mackerel in Japan (Yamashita 2010; Yamashita and Yamashita 2015). The meat appears “cooked” although raw, and the flesh lacks the typical bright red color of meat. The burnt meat has a more watery, softer texture, and is often seen in fish under stress conditions, for example those caught during the summer spawning period when the water temperature is high. The oxidative stress is caused by selenium deficiency and hypoxia (Tohfuku et al. 2021). After catching, extensive apoptosis and autophagy occur in the white muscle, and hemolysis occurs in fish that contain blood selenium levels of less than 1 µg/g tissue. The antioxidant effects of selenoneine may be essential in enabling fish to adapt and survive in low-oxygen marine environments. In rats, selenium deficiency was shown to induce hemolysis (Rotruck et al. 1972). White muscle disease in selenium-deficient animals shows similar features (Combs and Combs 1986). Selenium levels in the blood of bluefin tuna were closely related to the radical scavenging activity against red blood cells (Yamashita 2010). Therefore, selenium supplementation in cultured fish might reduce the burnt meat problem in aquaculture, and the nutritional selenium requirement of ocean fish species should be evaluated. In aquaculture, since several studies reported Se supplements for fish species using selenium yeast, selenomethionine, Se nanoparticles, and selenite (Ashouri et al. 2015; Cotter et al. 2008, Dawood et al. 2019; Elia et al. 2011; Fz et al. 2009; Han et al. 2011; Hatfield et al. 2006; Ilhan et al. 2016; Shahpar and Johari 2019; Wang et al. 2011), we used selenomethionine and sodium selenite in this study as control diets.

Selenoneine, 2-selenyl-*N*_*α*_, *N*_*α*_, *N*_*α*_-trimethyl-_L_-histidine, is a novel selenium containing compound found in the blood and other tissues of fish (Yamashita and Yamashita 2010; Yamashita et al. 2010, 2011, 2013a) and is a major organic selenium compound in fish as well as selenoproteins (Yamashita et al. 2013a). Selenoneine is taken up into cell by the specific organic cation/carnitine transporter-1 (OCTN1) (Yamashita et al. 2010) and binds to the heme of hemoglobin and myoglobin to prevent autooxidation of iron ions (Yamashita and Yamashita 2010). For industrial application of selenium, selenoneine is produced from fisheries waste and be used for functional foods and feed. Although the antioxidant function of selenoneine is expected, there are few studies on oral administration to fish. Red seabream *Pagrus major* is one of the most popular marine fish and its farming is active in Japan (Imai 2005). In Japan, wild catch of red sea bream was 9187 t in 2020, and aquaculture production was approximately 62,300 t in 2020. Thus, red sea bream is a popular marine fish species (https://www.e-stat.go.jp/en/stat-search/files?page=1&layout=dataset&toukei=00500208&tstat=000001015664&tclass1=000001036597&tclass2=000001164586&stat_infid=000032187641).

Although the color of the body surface is important for red seabream, cultured red seabream may turn black by oxidative stress such as sunlight. Selenoneine suppresses melanin synthesis by inhibiting tyrosinase (Seko et al. 2019). Hence, selenoneine may reduce oxidative stress conditions and melanin synthesis of cultured red seabream.

In this study, we compared the accumulation and antioxidant function of selenoneine and other selenium compounds, i. e., sodium selenite and selenomethionine, in the muscle and other tissues of red seabream.

## Materials and Methods

### Materials

Sodium selenite was purchased from Wako Pure Chemical Industries (Osaka, Japan). Seleno-_L_-methionine was purchased from Sigma-Aldrich Japan (Tokyo, Japan). Selenoneine was purified from internal organs of skipjack tuna and blood of tuna at the National Fisheries University according to methods described previously (Yamashita and Yamashita 2010).

Preparation of Feed The feed was purchased from Hayashikane Sangyo (Yamaguchi, Japan). Sodium selenite, selenomethionine, or selenoneine solution were sprayed on feed and then dried in vacuum. The final selenium concentration of each feed is shown in Table S1.

### Fish

Red seabream fry (average body weight of 5.4 g) was obtained from private seed and seedling production company (Oita, Japan) and cultured for seven months up to average body weight of 250 g. Chemical composition of control diet was containing crude protein 50%; crude fiber crude lipid 10.0%; Ca 2.3%; ash 16.0%; and phosphorus 1.5%. Each selenium-added diet was fed twice a day for 4 weeks in an amount of 1% of the average body weight. Body weight of fish in each tank was measured when fish were sampled and amount of diet was calculated according to the body weight of every sampling day. The added selenium concentrations in the diets were set in 0 ppm, 1 ppm, and 2 ppm selenium according to the previous study (Tohfuku et al. 2021). After that, they were fed a commercial diet twice a day for 1 week. The test groups were the control group, the sodium selenite group (1 mg Se/kg feeding group and 2 mg Se/kg feeding group), the selenomethionine group (1 mg Se/kg feeding group and 2 mg Se/kg feeding group), and the selenoneine group (1 mg Se/kg feeding group and 2 mg Se/kg feeding group) were set. The number of fish raised was 15 from the start of rearing in each tank until the 2nd week, 10 from the 3rd to 4th week, and 5 in the 5th week. An FRP circular tank (550 L capacity; 500 L seawater) was used as the tank, and the fish were reared under natural seawater (flow rate: 314 L/h). We reared red seabream by feeding of 1% dry pellet containing of sodium selenite, selenomethionine, or selenoneine of body weight twice a day for 4 weeks. After that, we replaced to 1% of commercial dry pellet of body weight twice a day for 1 week from the selenium supplementation. Average water temperature was 20.9 ℃ (19.0–23.9 ℃) and average dissolved oxygen was 7.41 mg/L. The body weight change of red seabream for 5 weeks is shown in Table S2.

Red seabream was anesthetized by adding 2 mL of anesthetic (1/100 volume of 2-methylquinoline dissolved in ethanol) to 1 L of seawater. A small amount of an anticoagulant (heparin sodium dissolved in physiological saline; 800 units/mL) was placed in a syringe in advance, and blood was collected from the caudal peduncle of each fish. The collected blood was centrifuged at 5000 rpm for 5 min (4 °C), divided into a plasma fraction and a precipitated fraction, and stored at − 50 °C. The precipitated fraction was used for analysis as the blood cell fraction.

As shown in Table S1, we used commercially available dry pellet as the control diet, and dry pellets containing selenium compounds, such as sodium selenite, selenomethionine, and selenoneine at 1 mg Se/kg or 2 mg Se/kg for feeding experiments.

We measured total selenium concentration in the tissues, such as, white muscle, heart, kidney, spleen, hepatopancreas, brain, and blood cell (Table 1) for each feeding group.

**Table 1:** Total selenium concentrations in the tissues of red seabream

	Total selenium content (mg Se/kg)						
	Control	Sodium selenite		Selenomethionine		Selenoneine	
		1 mg Se/kg	2 mg Se/kg	1 mg Se/kg	2 mg Se/kg	1 mg Se/kg	2 mg Se/kg
White muscle							
0 weeks	0.37 ± 0.03						
2 weeks	0.32 ± 0.03	0.39 ± 0.02^*^	0.40 ± 0.02^*^	0.34 ± 0.03^*^	0.38 ± 0.05	0.39 ± 0.05^*^	0.35 ± 0.04
4 weeks	0.35 ± 0.03	0.39 ± 0.01^*^	0.39 ± 0.03^*^	0.38 ± 0.05	0.41 ± 0.05^*^	0.40 ± 0.05^*^	0.43 ± 0.04^*^
5 weeks	0.34 ± 0.03	0.38 ± 0.06	0.36 ± 0.05	0.35 ± 0.04	0.37 ± 0.03^*^	0.38 ± 0.04^*^	0.38 ± 0.03^*^
Heart							
0 week	0.73 ± 0.12						
2 weeks	1.12 ± 0.14	1.05 ± 0.20	0.99 ± 0.10^*^	1.18 ± 0.12	1.23 ± 0.09	1.11 ± 0.11	1.32 ± 0.18^*^
4 weeks	1.10 ± 0.11	1.27 ± 0.11^*^	1.22 ± 0.08^*^	1.18 ± 0.17	1.26 ± 0.17^*^	1.16 ± 0.11	1.19 ± 0.09^*^
5 weeks	1.04 ± 0.08	1.20 ± 0.09^*^	1.04 ± 0.07	1.25 ± 0.15^*^	1.27 ± 0.13^*^	1.09 ± 0.15	1.28 ± 0.29^*^
Kidney							
0 week	1.73 ± 0.36						
2 weeks	2.17 ± 0.07	2.46 ± 0.31^*^	2.50 ± 0.19^*^	2.74 ± 0.47^*^	2.92 ± 0.32^*^	3.22 ± 0.18^*^	3.46 ± 0.43^*^
4 weeks	2.68 ± 0.32	3.07 ± 0.24^*^	3.06 ± 0.34^*^	2.93 ± 0.49	2.89 ± 0.44	3.17 ± 0.40^*^	4.03 ± 0.72^*^
5 weeks	2.56 ± 0.34	2.60 ± 0.32	2.71 ± 0.39	2.44 ± 0.24	2.39 ± 0.27	2.96 ± 0.32^*^	3.51 ± 0.49^*^
Spleen							
0 week	1.99 ± 0.48						
2 weeks	2.43 ± 0.51	2.58 ± 0.16	2.80 ± 0.25	2.55 ± 0.19	2.67 ± 0.38	3.45 ± 0.56^*^	4.69 ± 0.79^*^
4 weeks	2.89 ± 0.24	3.09 ± 0.47	2.94 ± 0.22	2.45 ± 0.30^*^	2.48 ± 0.35^*^	3.23 ± 0.37^*^	5.20 ± 1.01^*^
5 weeks	2.54 ± 0.24	2.80 ± 0.28^*^	2.47 ± 0.35	2.10 ± 0.32^*^	2.08 ± 0.21^*^	3.15 ± 0.83^*^	3.16 ± 0.68^*^
Hepatopancreas							
0 week	0.70 ± 0.11						
2 weeks	0.97 ± 0.14	1.12 ± 0.16	1.31 ± 0.15^*^	0.91 ± 0.08	1.03 ± 0.12	1.27 ± 0.18^*^	1.51 ± 0.26^*^
4 weeks	0.98 ± 0.06	1.40 ± 0.19^*^	1.45 ± 0.13^*^	1.13 ± 0.23	1.06 ± 0.22	1.22 ± 0.14^*^	1.35 ± 0.18^*^
5 weeks	1.02 ± 0.13	1.31 ± 0.23^*^	1.28 ± 0.18^*^	1.09 ± 0.07	1.33 ± 0.30^*^	1.34 ± 0.26^*^	1.40 ± 0.25^*^
Brain							
0 week	0.38 ± 0.08						
2 weeks	0.47 ± 0.05	0.49 ± 0.03	0.50 ± 0.03	0.45 ± 0.08	0.49 ± 0.14	0.48 ± 0.02	0.50 ± 0.04
4 weeks	0.40 ± 0.05	0.54 ± 0.07^*^	0.45 ± 0.10	0.45 ± 0.04^*^	0.42 ± 0.09	0.55 ± 0.06^*^	0.56 ± 0.05^*^
5 weeks	0.41 ± 0.08	0.35 ± 0.09	0.33 ± 0.11	0.45 ± 0.09	0.48 ± 0.06^*^	0.48 ± 0.09	0.47 ± 0.04
Blood cell							
0 week	1.71 ± 0.24						
2 weeks	2.12 ± 0.23	2.44 ± 0.27^*^	2.49 ± 0.29^*^	2.90 ± 0.32^*^	3.57 ± 0.35^*^	3.98 ± 1.00^*^	9.28 ± 1.67^*^
4 weeks	2.91 ± 0.62	3.11 ± 0.40	2.86 ± 0.17	2.69 ± 0.60	2.74 ± 0.25	5.93 ± 1.00^*^	9.94 ± 2.05^*^
5 weeks	3.03 ± 0.44	3.18 ± 0.76	3.13 ± 0.48	3.04 ± 0.42	3.01 ± 0.65	4.70 ± 0.53^*^	9.10 ± 1.48^*^

^*^Asterisks indicate statistical significance in compared with the control group (*p* < 0.05, ANOVA)

### Selenium Concentration Measurement

Total selenium concentration was determined by fluorometric assay with 2,3-diamino-naphthalene, after digestion in 1.5 mL of nitric acid and perchloric acid (1:2 in volume) at 200–220 ℃ (Watkinson 1966; Yamashita and Yamashita 2010).

### Selenoneine Concentration Measurement

Selenoneine was separated by chromatography using a Shodex GF-310 column (4.5 mm × 150 mm; Showa Denko, Tokyo, Japan) equilibrated with 100 mM ammonium formate buffer containing 0.1% (w/v) Igepal CA-630 (MP Biomedicals, CA, USA). The injection volume was at 10 μL. The mobile phase delivered at 0.5 mL/min isocratically. ^82^Se was detected with high performance liquid chromatography inductively coupled plasma mass spectrometry (HPLC-ICP-MS; Agilent 1100 Series and Agilent 7500 Series, Agilent Technology) (Yamashita and Yamashita 2010). During separation, selenoneine was eluted at a retention time of 200 s, and the selenium concentration was determined using selenoneine as a standard.

### Oxidation–reduction Potential (ORP) Measurement

ORP was measured with ORP electrode (9300, Horiba, Kyoto, Tokyo). The electrode was pierced into muscle and pressed gently until the potential stabilized for 60 s.

### Glutathione Peroxidase (GPx) Activity

GPx activity in the hepatopancreas of red seabream in each group 4 weeks after feeding with selenium supplementation diet was examined as a biochemical marker for selenium supplementation by monitoring the oxidation of nicotinamide adenine dinucleotide phosphate (NADPH) in the presence of glutathione reductase, which catalyzes the reduction of oxidized glutathione formed by GPx (Rotruck et al. 1973; Yamashita et al. 2012). The activity was measured at 37 ℃ in a solution containing 50 mM sodium phosphate buffer (pH 7.0), 2.5 mM EDTA, 1 mM sodium azide, 0.5 mM reduced glutathione, 0.005 U/mol glutathione reductase, 0.4 mM hydrogen peroxide, and 0.15 mM NADPH. Solution fluorescence was measured with a fluorometer at 460 nm with an excitation wavelength of 355 nm. One enzyme unit of GPx activity is defined as 1 µmol of NADPH oxidized per minute at 37 ℃.

### Uptake Factor

Uptake factor is defined as a ratio of selenoneine concentration in muscle or blood cells to those in feed, and is the same as the biomagnification factor (Yamada et al. 1994). Thus, uptake factor is an indicator showing the degree of accumulation of selenoneine in muscle or blood cells by dietary intake of feed. The uptake factor was calculated by the following equation on the equilibrium period of the dietary exposure experiments:

Uptake factor = ((Cen − Cb) / CF) × 10^–3^where Cen is selenoneine concentration in muscle or blood cells of fish fed with selenoneine-contained feed (ng/kg), Cb is selenoneine concentration in muscle or blood cells of the control fish (ng/kg wet), and CF is the selenoneine concentration in the contained feed (μg/kg).

The elimination half-life in muscle and blood cells was calculated using the total selenium concentration data of the 4th and 5th weeks after replaced to 1% of normal commercial dry pellet in Table 1 by the method described previously (Nomura et al. 2011).

### Statistical Analysis

The results are expressed as means ± standard error. Data were analyzed by Dunnett’s multiple comparison test to identify any significant differences (*P* < 0.05). For the Pearson correlation coefficient analysis, the GraphPad Prism 9 software (GraphPad Software, San Diego, CA, USA) was used. Statistical significance was set at *P* < 0.05.

## Results

### Total Selenium Concentration

In the group of the sodium selenite at 1 mg Se/kg and 2 mg Se/kg, total selenium was accumulated in white muscle, kidney, and hepatopancreas in compared with the control group. In the group of the selenomethionine at 1 mg Se/kg and 2 mg Se/kg, total selenium was accumulated in white muscle and heart. In the group of the selenoneine at 1 mg Se/kg, total selenium was accumulated in white muscle, kidney, spleen, hepatopancreas, brain, and blood cell. In the group of the selenoneine at 2 mg Se/kg, total selenium was accumulated in white muscle, heart, kidney, spleen, hepatopancreas, brain, and blood cell.

### Selenoneine Concentrations

Selenoneine concentrations were determined in the white muscle and blood cell by monitoring ^82^Se levels by HPLC-ICP-MS (Table 2). In the control group, selenoneine levels were 0.018 ± 0.001 mg Se/kg in the white muscle and 2.38 ± 0.71 mg Se/kg in the blood cells. In the fish fed with selenoneine at 1 mg Se/kg and 2 mg Se/kg, selenoneine was accumulated in both white muscle and blood cell. Selenonine levels were 4.72 ± 1.06 mg Se/kg in the blood cells and 0.025 ± 0.003 mg Se/kg in the white muscle of fish fed with 1 mg Se/kg, and 10.29 ± 3.14 mg Se/kg in the blood cells and 0.030 ± 0.007 mg Se/kg of fish fed with 2 mg Se/kg. These selenoneine concentrations accounted for more than 63% of total selenium in blood cell and for 4.6–7.0% of selenium in white muscle. In contrast, the fish fed with other selenium compounds, sodium selenite, and selenomethionine contained small amount of selenoneine similar to the control fish.

**Table 2:** Selenoneine concentration in white muscle and blood cell in the fish cultured for 4 weeks

Tissue	Selenoneine concentration (mg Se/kg)						
	Control	Sodium selenite		Selenomethionine		Selenoneine	
		1 mg Se/kg	2 mg Se/kg	1 mg Se/kg	2 mg Se/kg	1 mg Se/kg	2 mg Se/kg
White muscle	0.018 ± 0.001	0.019 ± 0.002	0.020 ± 0.003	0.019 ± 0.003	0.019 ± 0.003	0.025 ± 0.003^*^	0.030 ± 0.007^*^
Blood cell	2.38 ± 0.71	1.98 ± 0.39	1.91 ± 0.43	1.64 ± 0.34	1.78 ± 0.34	4.72 ± 1.06^*^	10.29 ± 3.14^*^

^*^Asterisks indicate statistical significance in compared with the control group (*p* < 0.05, ANOVA)

We evaluated the uptake factor and the half-life from the concentrations of selenoneine in the feed, white muscle, and blood of the fish cultured for 4 weeks and after replaced from selenoneine-feed to control feed for additional 1 week. The uptake factor of selenoneine from the artificial feed containing selenoneine at 0.1–2 mg Se/kg was calculated to be 0.0062 in the white muscle and 4.0 in the blood, indicating that the uptake in the blood cells was higher than that in white muscle (Table 3). In addition, the elimination half-life of selenium in blood cells and white muscle was estimated from Table 1. The half-life of total selenium in the blood cells and white muscle were estimated to be 60 days in the white muscle and 30 days in the blood (Table 1).

**Table 3:** Selenoneine concentration in white muscle and feed, and uptake factor in selenoneine 1 mg Se/kg and 2 mg Se/kg administration group

	Control	Selenoneine 1 mg Se/kg	Selenoneine 2 mg Se/kg
Total Se concentration in feed (mg Se/kg)	1.9	3.0	4.1
Selenoneine concentration in feed (mg Se/kg)	0.1	1.1	2.2
Selenoneine concentration in white muscle (µg Se/kg)	17.7 ± 1.1	25.4 ± 3.2	30.0 ± 6.6
Uptake factor		7.2 ± 2.7	5.6 ± 2.7

#### ORP

**Fig. 1:**
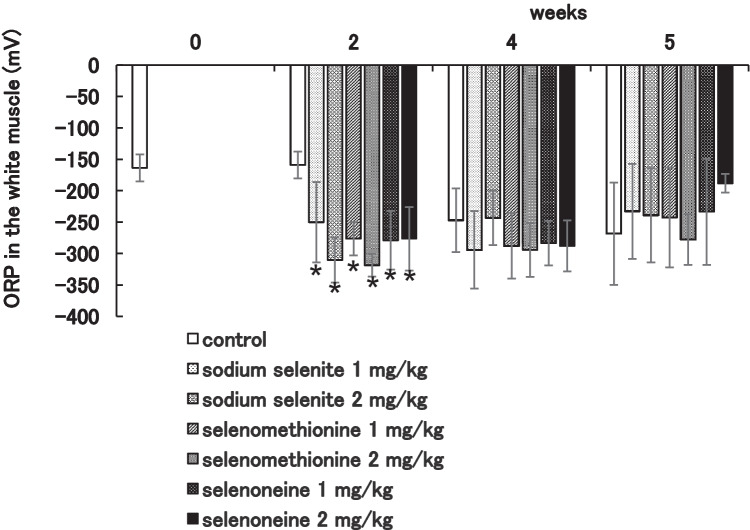
Oxidation–reduction potential (*P* < 0.05). Red seabream were fed dry pellet added each selenenium compound 1 or 2 ppm for 4 weeks and then fed dry pellet for a week. ORP was measured in white muscle

**Fig. 2:**
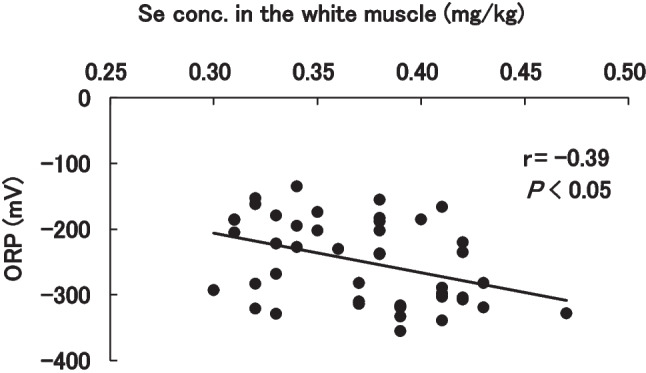
Relationship between total selenium content and ORP in white muscle

ORP in muscle was analyzed to evaluate the redox status in fish (Fig. 1). All of selenium administration groups decrease ORP at 2 weeks compared to control, but not 4 weeks and 5 weeks. Correlation coefficient between total selenium content and ORP in white muscle was − 0.46 (Fig. 2), and the *t*-test showed a significant (*P* = 0.032).

### GPx Activity

**Fig. 3:**
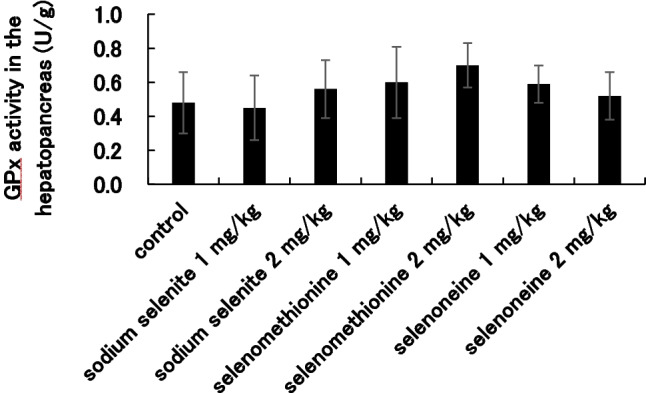
GPx activity in hepatopancreas at 4 weeks

GPx activity in hepatopancreas of the fish at 4 weeks were compared with the activities of the selenium supplemented groups (Fig. 3). There was no apparent difference in the activities among the different groups.

## Discussion

A feeding experiment of red seabream with feeds containing of sodium selenite, selenomethionine, or selenoneine was carried out for 4 weeks. After selenium supplementation, we replaced to normal commercial dry pellet for 1 week from the selenium supplementation, and tissue distribution of total selenium was determined. Selenium supplementation with selenoneine, selenomethionine, and sodium selenite enhanced selenium accumulation in the white muscle, kidney, and hepatopancreas in comparison with the control group. By the dietary intake of selenoneine, total selenium concentrations were increased in the white muscle, heart, kidney, spleen, hepatopancreas, brain, and blood in a dose-dependent manner during the trials after 2 weeks. Dietary intake of selenoneine as well as sodium selenite and selenomethionine reduced ORP.

Levels of selenium were determined in white muscle, heart, kidney, spleen, hepatopancreas, brain, and blood cell at 2 weeks, 4 weeks, and 5 weeks. Levels of selenoneine were determined in white muscle and blood cell at 2 weeks. Dietary intake of sodium selenite or selenomethionine resulted in accumulation selenium in some tissue while dietary intake of selenoneine resulted in accumulation selenium in all tissue we measured in this study. Especially, high levels of selenoneine accumulated in blood cell (Table 2). It is suggested that selenium accumulation mechanism differs for each selenium compound and selenoneine accumulates in each tissue through blood cell. We found that selenoneine was not produced from other selenium compounds, such as sodium selenite and selenomethionine in the red seabream (Table 2), which suggest that selenoneine might be accumulated by dietary intake from feeds but not biosynthesis from other selenium source. We also measured the elimination half-life of total selenium in the blood cells and white muscle were estimated to be 60 days in the white muscle and 30 days in the blood (Table 1), indicating that accumulated selenoneine in the blood cells and white muscle was eliminated gradually.

Selenoneine has antioxidant function (Yamashita and Yamashita 2010) and methylmercury (MeHg) detoxification (Yamashita et.al. 2013b). In a zebrafish toxicity assay, selenoneine demethylated MeHg and then the demethylated MeHg is taken up into secretory extracellur lysosomal vesicles and discharged through OCTN1 (Yamashita et.al. 2013b). Yamashita et.al. also reported red blood cells express the OCTN1 and accumulated selenoneine (Yamashita et.al. 2013a). Therefore, the reason why selenium accumulates in blood cells at a high level is that it exerts an antioxidant and mercury detoxifying functions on blood cell which have a high risk of oxidation and contain a large amount of methylmercury.

The ratio of selenium used in selenoneine in white muscle of wild red seabream was about 10% (Yamashita et al. 2011). In this study, that of the control and selenoneine 2 ppm administration groups were about 5% and about 7.0%, respectively. This result indicates that dietary intake of selenoneine increase the ratio of selenoneine and enhance nutritional status.

Selenoneine administration groups reduced ORP as well as sodium selenite and selenomethionine at 2 weeks. ORP in the control group at 4 and 5 weeks decreased, but it may be due to selenium originally in the commercial dry pellet. Our unpublished research showed that dietary intake of selenoneine in yellowtail reduced ORP and approached the ORP of wild fish. Measurement of ORP can be used for evaluation of antioxidant activity in the tissues by dietary intake of selenium in vivo.

In conclusion, we showed that selenoneine accumulated in the body and enhanced antioxidant function by dietary intake of selenoneine. To utilize selenoneine in food and aquaculture, we should demonstrate biological function in vivo and required amount of selenoneine for each fish species.

### Supplementary Information

Below is the link to the electronic supplementary material.

Supplementary file1 (DOCX 32 KB)

## Data Availability

All data generated or analyzed during this study are included in this published article.

## Abbreviations

ORP: Oxidation-reduction potential
HPLC-ICP-MS: High performance liquid chromatography inductively coupled plasma mass spectrometry
GPx: Glutathione peroxidase
GPC: Gel permeation chromatography
NADPH: Nicotinamide adenine dinucleotide phosphate
OCTN1: Organic cations/carnitine transporter-1
Se: Selenium
MeHg: Methylmercury
