# Supplementary material for: Nutritional Supplementation and Enhanced Antioxidant Function by Dietary Intake of Selenoneine and Other Selenium Compounds in Red Seabream Pagrus major
Source: Mar Biotechnol (NY). 2023 Jul 18;25(5):683–90. doi: 10.1007/s10126-023-10215-6 (PMC10665234; doi:10.1007/s10126-023-10215-6)
Supplement: Supplementary file 1 — Supplementary file1 (DOCX 32 KB) [file 10126_2023_10215_MOESM1_ESM.docx]

Table. S1 Total selenium concentration in dry pellet for feeding experiment

| diet | Total Se concentration (mg Se/kg) |
| --- | --- |
| control | 1.92 |
| sodium selenite (1 mg Se/kg) | 3.07 |
| sodium selenite (2 mg Se/kg) | 4.11 |
| selenomethionine (1 mg Se/kg) | 2.81 |
| selenomethionine (2 mg Se/kg) | 4.07 |
| selenoneine (1 mg Se/kg) | 2.98 |
| selenoneine (2 mg Se/kg) | 4.11 |

**Table S2** Growth of red seabream during the feeding experiment for 5 weeks

| feeding period | body weight (g) | | | | | | |
| --- | --- | --- | --- | --- | --- | --- | --- |
|  | control feed | selenium supplementation | | | | | |
|  |  | sodium selenite | | selenomethionine | | selenoneine | |
|  |  | 1 mg Se/kg | 2 mg Se/kg | 1 mg Se/kg | 2 mg Se/kg | 1 mg Se/kg | 2 mg Se/kg |
| before experiment  efore experiment | 250 ± 23 |  |  |  |  |  |  |
| 2 weeks | 316 ± 61 | 296 ± 46 | 290 ± 48 | 313 ± 52 | 309 ± 32 | 275 ± 56 | 338 ± 39 |
| 4 weeks | 339 ± 26 | 359 ± 50 | 351 ± 62 | 316 ± 22 | 333 ± 33 | 321 ± 47 | 378 ± 58 |
| 5 weeks | 335 ± 36 | 316 ± 55 | 363 ± 72 | 307 ± 65 | 322 ± 45 | 308 ± 52 | 394 ± 61 |
